# Novel Paradigm for Constructing Masses in Dempster-Shafer Evidence Theory for Wireless Sensor Network's Multisource Data Fusion

**DOI:** 10.3390/s140407049

**Published:** 2014-04-22

**Authors:** Zhenjiang Zhang, Tonghuan Liu, Wenyu Zhang

**Affiliations:** Department of Electronic and Information Engineering, Key Laboratory of Communication and Information Systems, Beijing Municipal Commission of Education, Beijing Jiaotong University, Beijing 100044, China; E-Mails: 11120129@bjtu.edu.cn (T.L.); 13120179@bjtu.edu.cn (W.Z.)

**Keywords:** mass, Dempster-Shafer evidence theory, Mahalanobis Distance, WSN

## Abstract

Dempster-Shafer evidence theory (DSET) is a flexible and popular paradigm for multisource data fusion in wireless sensor networks (WSNs). This paper presents a novel and easy implementing method computing masses from the hundreds of pieces of data collected by a WSN. The transfer model is based on the Mahalanobis distance (MD), which is an effective method to measure the similarity between an object and a sample. Compared to the existing methods, the proposed method concerns the statistical features of the observed data and it is good at transferring multi-dimensional data to belief assignment correctly and effectively. The main processes of the proposed method, which include the calculation of the intersection classes of the power set and the algorithm mapping MDs to masses, are described in detail. Experimental results in transformer fault diagnosis show that the proposed method has a high accuracy in constructing masses from multidimensional data for DSET. Additionally, the results also prove that higher dimensional data brings higher accuracy in transferring data to mass.

## 1. Introduction

Multi-sensor data fusion is a technology that makes it possible to combine information from multiple sources to obtain a unified picture [1]. In wireless sensor networks (WSNs), data fusion is a useful way to decrease or eliminate the uncertainty of decisions when dealing with information from different sources. It is widely used in state estimation problems [2], pattern recognition [3], robotics [4], and medical imaging [5]. Different theories have been proposed in multisource data fusion, such as the Bayesian approach, Dempster-Shafer evidence theory (DSET) [6], fuzzy set theory [7], and the rough set theory [8].

**Figure 1.**
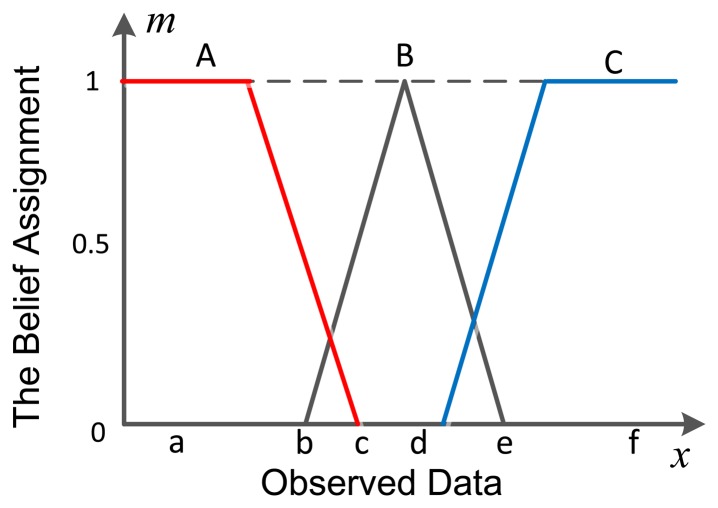
Common method used to transfer observed data into a belief assignment.

In a WSN, hundreds of pieces of data with different properties are collected by the nodes. To achieve a reasonable result, the theory used in this application should be good at transferring these large amounts of data with different properties into a unified result. DSET is an efficient way to deal with the uncertainty and imprecision of information [9], and its fusion framework has an advantage of combining different information into one, which makes it become a flexible method in WSN multisource information fusion. The mass function, also called basic belief assignment (BBA) function, is a prerequisite for using DS theory in reality. However, there are no fixed models to get mass in DSET. Hence how to use the hundreds of multisource pieces of data to construct the mass for DSET is the first problem that should be solved. A good and efficient paradigm for constructing an evidence structure must be set up because it is vital to get accurate conclusions from the information we collected. Suppose there is a classification problem with three possible results, the commonly used belief assignment transferring method is as shown in Figure 1.

In Figure 1, The X axis stands for the observed data and the Y axis is the belief assignment. The three possible patterns are A, B and C. The observed data is transferred to the belief assignment (mass) according to the intervals to which it belongs. This method is easy to implement, but its accuracy is low. The reason involves two aspects: (1) the method ignores the statistical features of the observed data. The mean value and the standard deviation are always different, except for their distribution intervals. Even though the sample data sets of A and B distribute the same interval, their statistical features are still different. In this perspective, this common way isn't able to get the correct mass of the observed data; (2) the observed data is always organized in a multi-dimensional pattern. For example, a sensor can monitor temperature and humidity at the same time, the data will be presented as (*T*,*H*), where *T* and *H* are temperature and humidity, respectively. How to calculate the belief assignment from multi-dimensional data becomes another problem. These two problems are why we develop the proposed method to transfer multi-dimensional data to mass for DSET.

In this paper, Mahalanobis distance (MD) is used to measure the similarity between an object and a class. A long MD corresponds to a low belief assignment, and a short MD means a large belief assignment. Unlike Euclidean distance (ED), MD indicates the “distance” of the data's covariance. It is not affected by the dimension of the data and is a more scientific measurement of the similarity between an observed object and a class than Euclidean distance, because MD considers the difference of the samples' statistical features, including the mean value and covariance. The two main problems existing in the common method will be solved by transferring the MD to mass. Besides, for a compound class, which means the mixed class of the power set in DSET, the masses can also be calculated using MD. The main process of the proposed method includes three main steps: firstly is the calculation of the intersection classes of the power set and then is the step calculating the MD between the object and the subset samples, the last step is the algorithm mapping MDs to masses. The experimental results will be described to verify the performance of the proposed method. The proposed algorithm is used in transformer fault diagnosis to construct masses of diagnosis evidences from data collected in the transformer's inner space. The obtained results prove that the proposed has a high accuracy in constructing masses for DSET, especially in high dimensional data.

The remainder of this paper is organized as follows: Section 2 illustrates the related work. In Section 3, the MD method and DSET are briefly introduced, and then the mechanism for transforming data into masses in DSET by using the MD-mass method is developed in Section 4. Section 5 depicts the scheme of the implementation process for the proposed algorithm. In Section 6, the experiment for transformer fault diagnosis is described, along with its results. Finally, the discussion and conclusions are presented in Section 7.

## 2. Related Work

Mahalanobis distance (MD) is a useful method to calculate the similarity of different samples [10]. It is used in many fields, including statistics [11], pattern recognition [12], and manufacturing control [13]. In this paper, we focus on the research of belief transferring model. Aside from the common method in Section 1, many belief assignment functions have been developed to obtain masses from observed data and they are proved to be reasonable in certain applications. Chakeri developed a method based on Fuzzy C-means to gain masses [14]; the method is good at obtaining belief assignments from imprecise information. Szlzenstein put forth an iterative estimation method based on Gaussian model [15]. In [16], a scheme for constructing an evidence structure that uses an artificial neural network (ANN) is proposed; the method is good at dealing with large scale data in applications like image processing. In [17], Xin developed three methods to construct the BBA function. These methods are based on gray correlation analysis, fuzzy sets, and attribute measure, respectively. They are proved to be reasonable in converting different data sources into masses. Other efforts have been made to solve this issue by using different methods and theories, like fuzzy entropy [18], automatic thresholding [19], and so on.

The research on BBA function can be summarized as follows: (1) different transfer functions are developed counter to different specific applications, like pattern recognition, image processing. There is no a unified framework suitable under all conditions; (2) the existing developed methods are not suitable in WSN multisource data fusion because they ignore the importance of statistical features, which is good for obtaining a more correct belief assignment; (3) many of them can't be implemented in sensor nodes because their complex computational process, such as the ANN method.

## 3. Preliminaries

In this section, the basic theories related to the proposed method will be introduced, including the DSET, the Closed World Assumption and the Open World Assumption and Mahalanobis distance.

### 3.1. DSET

DSET [20] is an extension of the classical probability theory. It is a good strategy to deal with the conflicts and imprecision in multisource data fusion. Given an object X, let Ω = (*ω*_1_,…, *ω_c_*) be the set composed by all possible results of X, where the classes in Ω must be mutually exclusive and exhaustive. Ω is called the frame of discernment of X, and 2^Ω^ is the power set of all possible subsets of Ω. The mass function of 2^Ω^ is defined as a function m: 2^Ω^ → [0,1], which satisfies the condition:

(1) $$\sum_{A\subseteq \Omega }m(A)=1\;\text{and}\;m(\emptyset )=0$$

where Ø denotes the null set, and m(A) is called the basic belief assignment of A, where A is a subset of Ω. The numerical value of m(A) represents the degree of trust of exact set A. Subset A with non-zero mass is called a focal element. The structure composed of focal elements and their masses is called an evidence structure, expressed as:

(2) {(A,m(A))|A⊆Ω,m(A)>0}

We call (*A*, *m*(*A*)) a piece of evidence. There are two types of evidences: singletons and compound sets. The above process is the step of representing evidence by using focal elements.

In DSET, the impact of evidences on proposition A has two points: belief and plausibility. They are defined as follows:

(3) $$\text{Bel}(A)=\sum_{B\subseteq A}m(B)$$

(4) $$\text{Pls}(A)=\sum_{A\cap B\neq \emptyset }m(B)=1-\text{Bel}(\bar{A})$$

where Bel(*A*), Pls(*A*) and (*Ā*) denote the belief function, plausibility function, and dubiety function, respectively. It is apparent that Bel(*A*) ≤ Pls(*A*) is satisfied in all situations. If Bel(*A*) = Pls(*A*), then A must be a singleton class. A focal element of DSET provides an explicit measure by a belief interval: [Bel(*A*), Pls(*A*)], where the lower and upper probabilities depict the belief and plausibility, respectively.

After we get the evidence structure, a rule of combination can be used to fuse all the independent evidences into one. The Dempster combinational rule is expressed as:

(5) $${⊕}_{i=1}^{n}m_{i}(A)=\frac{1}{1-K}\sum_{\cap_{i=1}^{n}A_{n}=A}\prod_{i=1}^{n}m_{i}(A_{i})$$

(6) $$\text{with}\;K=\sum_{\cap_{i=1}^{n}A_{n}=\emptyset }\prod_{i=1}^{n}m_{i}(A_{i})$$

where ⊕ is the symbol of the combination operator. *A_i_* designates the focal element of data source *i*. *K* indicates the conflict among the sources to be combined. After combining, a Pignistic probability can be made using the following expression [21]:

(7) $$\text{Bet}P(A_{i})=\sum_{A_{l}\subseteq A_{M}}\left(\frac{1}{\left|A_{M}\right|})m(A_{M}\right)$$

Bet*P*(*A_i_*) is called the Pignistic probability transformed by the final evidence structure. Then, a decision can be made by choosing the class with maximum Pignistic probability as the result of the fusion process.

### 3.2. Closed World Assumption and Open World Assumption

When a proposition's genuine nature is uncertain, the Closed World Assumption regards this proposition as a false proposition; in contrast, the Open World Assumption takes this proposition as an unknown proposition. For example, under the known condition “Juan is a Boston citizen,” we can make proposition A: “Juan is a citizen of New York.” From the viewpoint of the Closed World Assumption, A is false, while the Open World Assumption regards A as an unknown proposition, because Juan maybe a New York citizen, though the possibility is low.

In short, the Closed World Assumption is applicable in an environment where all the conditions are known to us. When there are unknown conditions, we can take the Open World Assumption. In DSET, for a null set, its mass must equal 0, and it belongs to the Closed World Assumption. In the Transferable Belief Model (TBM) [21], *m*(Ø) > 0 is allowed, and it agrees with the Open World Assumption. TBM extends the scope of using DSET, and our mass allocation strategy can also be divided into the Closed World Assumption and Open World Assumption.

### 3.3. Mahalanobis Distance

Let *X* be a data matrix (*n* × *p*), containing *n* objects measured by *p* variables. *X̅* (1 × *p*) is the column vector of every object's mean value. *σ* is the variance and *ρ* denotes the Pearson correlation coefficient. Then, a variance–covariance matrix of *X* can be expressed as:

(8) $$C_{X}=\left[\begin{array}{cccc} \sigma_{1}^{2} & \rho_{12}\sigma_{1}\sigma_{2} & \cdots & \rho_{1n}\sigma_{1}\sigma_{n}\\ \rho_{21}\sigma_{2}\sigma_{1} & \sigma_{2}^{2} & \cdots & \rho_{2n}\sigma_{2}\sigma_{n}\\ \vdots & \vdots & \ddots & \vdots \\ \rho_{p1}\sigma_{p}\sigma_{1} & \rho_{p2}\sigma_{p}\sigma_{2} & \cdots & \sigma_{n}^{2} \end{array}\right]$$

with:

(9) $$\rho_{kl}=\frac{E(x_{k}x_{l})-E(x_{k})E(x_{l})}{\sqrt{E(x_{k}^{2})-E^{2}(x_{k})}\sqrt{E(x_{l}^{2})-E^{2}(x_{l})}}$$

where *x_k_* and *x_l_* are objects in *X* with subscript *k* and *l*. $C_{X}^{-1}$ is the inverse matrix of *C_X_*, Mahalanobis distance of object *x_o_*(1×*p*) to *X* is:

(10) $$MD(x_{o},X)=\sqrt{{\left(x_{o}-\bar{X}\right)}^{T}C_{X}^{-1}(x_{o}-\bar{X})}$$

**Figure 2.**
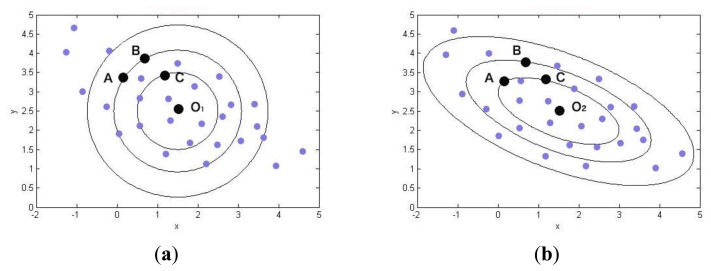
Difference between ED and MD in metric distance. (**a**) Distance defined by ED. (**b**) Distance Defined by MD.

We can see that the MD method is a way to calculate the similarity of two objects by their covariance. To get a better understanding of MD, a figure can be depicted as shown in Figure 2. The distributed points are sample points and their center points are O_1_ and O_2_. From the viewpoint of ED, the circles represent equal EDs to center point O_1_. Therefore, we know that point A and point B are equal to center point O_1_, because they have the same ED to O_1_. Things will be changed in (b), where the circles stand for equal MDs to center point O_2_. Unlike ED, MD is not the spatial distance but the distance of covariance. Thus, point A and point C are the same to center point O_2_. In reality, the distribution of objects is never a “circle,” but is more like a kind of ellipse. Apparently, MD is a more accurate and effective metric for the similarity than ED.

## 4. MD-mass Method Process

The process of the proposed method includes three main steps. The first is classifying the compound sample classes of the power set. Next step is calculating the MDs from new observed objects to all subsets. Then the obtained MDs will be transferred to the masses in step 3.

### 4.1. Calculation of Intersection Classes' Scope

It is easy to calculate the MD between an object and a singleton (crisp) class, but we can't calculate the MD between an object and a compound class directly. At the beginning, an original data sample belongs to a singleton class, but not to a compound (or mixed) class. One of the great advantages of DSET is that a certain degree of imprecision and conflicts between evidences are allowed to exist, and DSET is good at dealing with this issue. Thus, the method used to obtain the samples of the compound classes is very important.

#### 4.1.1. The Calculation with One Dimensional Data

For one dimensional data, the intersection classes are easy to find out. Given two sample sets *X* = {*x*_1_, *x*_2_,…*x_n_*}, *Y* = {*y*_1_, *y*_2_,…*y_n_*}, which have been preprocessed and the abnormal data have been removed. We set *I_X_* is the interval that contains all possible elements of *X* and the scope of the elements in *I_X_* are all in the interval [*X*_min_, *X*_max_]. It is the same with *I_Y_*. The intersection interval is set as:

(11) $$\begin{array}{c} I_{XY}=I_{X}\cap I_{Y}\\ \text{with}\forall z_{k}\in I_{XY},Y_{\min}\leq z_{k}\leq X_{\max} \end{array}$$

**Figure 3.**
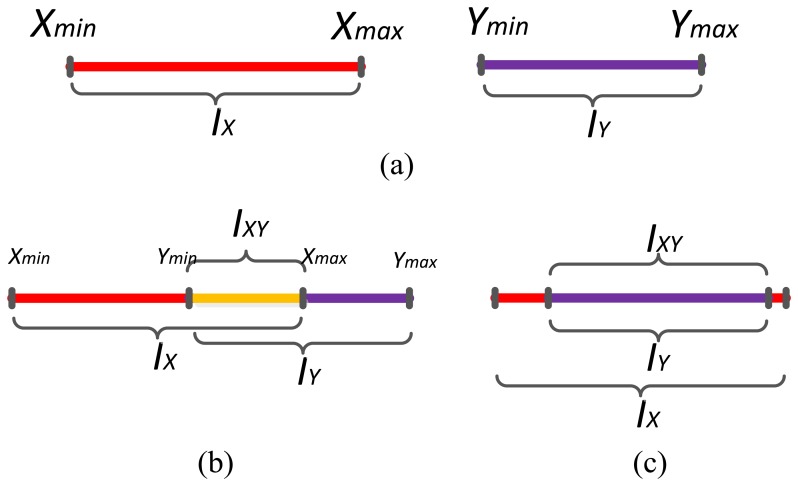
Calculation of intersection scope with one dimensional data. (**a**) Intersection interval of X and Y is null set. (**b**) Intersection interval of X and Y. (**c**) Intersection interval of X and Y when Y's interval is contained in X.

The intersection class is shown in Figure 3. In Figure 3a, there is no intersection interval between *I_X_* and *I_Y_*, thus their intersection is null set. In Figure 3b, the intersection is *I_XY_* and its interval is [*Y*_min_, *X*_max_]. If an element of *I_X_* or *I_Y_* belongs to the interval [*Y*_min_, *X*_max_], it belongs to *I_XY_*, too. In (c), *I_Y_* is contained in *I_X_*, so all elements of *I_Y_* and the elements in *I_X_* in the interval [*Y*_min_, *Y*_max_], belong to *I_XY_*. In reality, the data in different situation always distribute in different intervals with different statistical features. Here it must be emphasized that the statistical features of the samples are not shown in the figure. Though *I_Y_* and *I_XY_* are the same intervals in Figure 3c, sample sets of *I_Y_* and *I_XY_* are different. For example, the sample set in *I_Y_* has 100 samples, the mean value and variance are 10, 2.5, respectively. While sample set in *I_XY_* has more than 100 samples, the mean value and variance are 15, 2.0, respectively. In this situation, their statistical features, like mean value and standard deviation, are different too. They still can be classified because the MDs of an object to their sample data are different.

#### 4.1.2. The Calculation with 2 or Higher Dimensional Data

The MD between an object and different samples can be calculated in different dimensions. Thus, if the observed data are in multi-dimension, their intersection scope is also in the corresponding dimension. Here the intersection scope is also defined as:*I_AB_* = *I_A_* ∩ *I_B_*.

**Figure 4.**
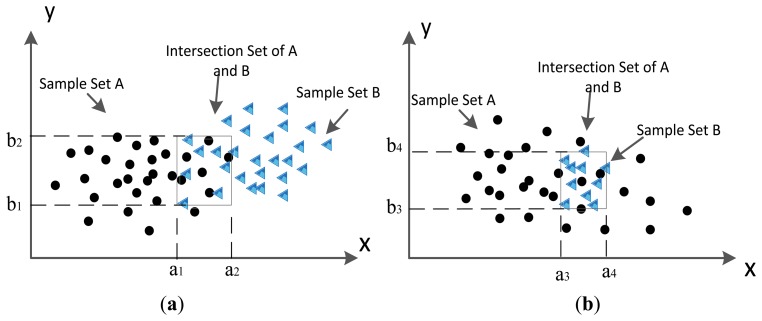
Calculation of intersection scope with 2-dimensional data. (**a**) Intersection scope of A and B. (**b**) Intersection scope of A and B when B's scope is contained in A.

If an object distributes in the scope of *I_AB_*, it belongs to the corresponding intersection sample set. In 2-dimension space, the intersection classes' scope is shown in Figure 4, which describes the way to find intersection class between sample set A and sample set B in a 2 dimension space. The black dots stand for elements of set A and the blue triangles denote the elements of set B. Let *I_A_* be the set that contain all possible elements of A. It is the same with *I_B_*. In Figure 4a of, the compound class *I_AB_* is the intersection of *I_A_* and *I_B_*, it is the same with Figure 4b. The obtained intersection scope in (a) is ([a_1_, a_2_], [b_1_, b_2_]), in Figure 4b, it is ([a_3_, a_4_], [b_3_, b_4_]). The sample set of *I_AB_* are comprised by the samples that distribute in the scope of *I_AB_*. The difference is that in Figure 4b, *I**_AB_* = *I_B_* ⊂ *I_A_*. The sample set of *I_AB_* are comprised by all samples of B and partial samples of A that belong to scope of *I_AB_*. In this situation, their distribution scopes are the same, but their statistical features are not equal to each other, because the samples they included are different.

In 3-dimensional space or even higher dimensional space, the intersection space is calculated as the same way as 2-dimensional space. Generally speaking, the higher dimension brings higher distinguishability.

### 4.2. Calculation of Evidences' MDs

Given Ω = (*ω*_1_,…,*ω_c_*) as the frame of discernment and *t* is the object to be classified. *M_j_* is the sample data of a subset in 2^Ω^, except the null set. $\bar{M_{j}}$ is the column vector of every sample mean value. For object *t*, we can get the MD between *t* and *M_j_* by the expression:

(12) $$MD(t,M_{j})=\sqrt{{\left(t-\bar{M_{j}}\right)}^{T}C_{M_{j}}^{-1}(t-\bar{M_{j}})},j=1,2,\ldots ,2^{c}-1$$

where *MD*(*t*, *M_j_*) denotes the MD of *t* to subset *M_j_*, and *C_Mj_* is the variance-covariance matrix of *M_j_*, *c* is the number of elements in the frame of discernment. The samples of *M_j_* are very important because they are the standard for whether an object belongs to *M_j_* or not.

### 4.3. Mapping MDs to Masses

In this paper, we take MDs as the basis of the basic belief assignments of the evidences. Now, a mechanism should be set up to map MDs to masses. This mechanism must satisfy the following principles:

(1) Every subset should get a reasonable mass in order to conduct the fusion process by DSET.
(2) The sum of all the masses must equal 1, and any evidence's corresponding mass should be in the range [0,1].
(3) The mass function should be a monotone decreasing function, which means the mass decreases with increasing MD.

In a neural network, there are several types of common transfer functions, like logsig and tansig [22]. Here, we use logsig as the mapping function to convert the MDs to masses. To subset *A*, the assigned mass can be calculated by:

(13) $$m(A|MD_{A})=f(MD_{A})$$

(14) $$\text{with}\;f(MD_{A})=1-\frac{1}{1+e^{-k(MD_{A}-\mu )}}$$

where m(*A*|*MD_A_*) is the assigned mass of subset *A* with *MD_A_*, *f*(*MD*)*_A_* is a monotonically decreasing transfer function converting MD between object and subset *A* to evidence's mass. *μ* is the mean of the MDs, *k* is the adjustment coefficient, and the shape of the function will be changed when *k* changes its value.

**Figure 5.**
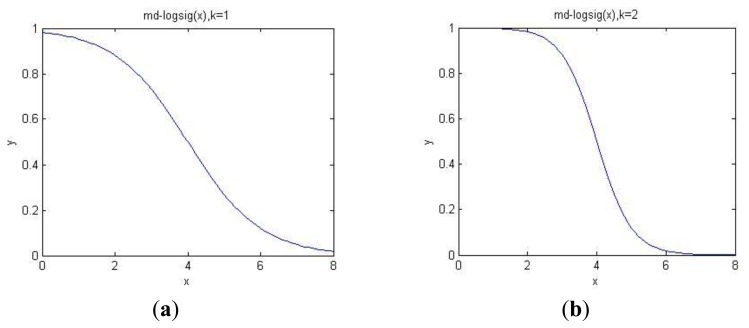
Curve of transfer function with different k. (**a**) k = 1. (**b**) k = 2.

Figure 5 shows the curve of the transfer function, in Figure 5a, *μ* = 4 and *k* = 1, in Figure 5b, *μ* = 4 and *k* = 2. Horizontal axis is calculated MD, vertical axis is the transferred mass. According to this function, when MD < 2, the mass of the corresponding evidence is close to 1, whereas in the interval [2,6], the mass will decrease as the MD increases. When *k* = 2, the curve is steeper than the line of *k* = 1. Thus, we should adjust the value of *k* according to the actual situations to guarantee the transferring accuracy as high as possible.

The transfer function satisfies the principle of mass assignments we just proposed. When the MD between an object and a class is less than a certain value (threshold value), it belongs to the class with a high probability. If MD exceeds the threshold value, the probability decreases with the increasing of MD's value. When MD is larger than another certain value, the probability is quite low and is virtually zero. For example, when we judge whether a man is middle aged or not, if he is 40–50, we can be sure that he belongs to the middle age class. If his age is 30–40 or 50–60, the boundaries are not clear because there is a possibility that he is a youth or an old man. In this situation, the probability of middle age will decrease when the MD of his age increases. If his age is younger than 30 or older than 60, we can be sure that he is not a middle-aged man; in this case, the probability is very low.

### 4.4. Automatic Adjustment of k

In the previous section, we showed that *μ* is the mean value of the MDs between an object and a class of 2^Ω^. Thus, we know its value by computing the mean of the MDs quickly and easily. The main problem is how to adjust the value of *k* automatically and properly.

Let *σ* be the standard deviation of the calculated MDs. According to the Central Limit Theorem of statistics, and we can assume that the original data agrees with the Gaussian distribution. In a Gaussian distribution, the original data complies with the “3*σ* principle”.

In a Gaussian distribution, *σ* denotes the standard deviation and *μ* denotes the mean value. The probability that a value is in the interval (*μ* − *σ*, *μ* + *σ*) is 0.6826. The probabilities are 0.9544 and 0.9974 for intervals of (*μ* − 2*σ*, *μ* + 2*σ*) and (*μ* − 3*σ*, *μ* + 3*σ*), respectively. Thus, we can suppose that almost all the data in the Gaussian distribution belongs to the interval (*μ* − 3*σ*, *μ* + 3*σ*), and the probability that a value will exceed the interval is not larger than 0.3%.

We set *λ*_max_ to be the upper threshold value of the output masses and *λ*_min_ as the lower threshold value. Apparently, *λ*_max_ is the belief assignment when MD equals to 0 and *λ*_max_ should approximate to 1:

(15) $$\begin{array}{cc} \lambda_{\max}=1-\frac{1}{1+e^{k\mu }}, & x=0 \end{array}$$

*λ*_min_ is the belief assignment when MD is larger than (*μ* + 3*σ*), according to the “3*σ* principle”, *λ*_min_ should approximate to 0.

(16) $$\begin{array}{cc} \lambda_{\min}=1-\frac{1}{1+e^{-k•3\sigma }}, & x-\mu =3\sigma \end{array}$$

Expressions (15) and (16) can be modified as:

(17) $$k=\max\{\frac{1}{\mu }[\ln(\lambda_{\max})-\ln(1-\lambda_{\max})],\frac{1}{3\sigma }[\ln(1-\lambda_{\min})-\ln(\lambda_{\min})]\}$$

*λ*_min_ and *λ*_max_ can be set in the interval [0.001, 0.003] [0.995, 0.999], respectively.

## 5. Scheme for Constructing Masses in DSET

In Section 4, the process of using MD to realize the masses in DSET was developed. The main idea of the algorithm is to construct the basis of the basic belief assignments through the prepared samples. Then, the MDs between the object and the samples are computed, and the following step is mapping the MDs to masses. Based on a real situation, we can choose a closed or open world. Finally, the output masses should be normalized.

**Figure 6.**
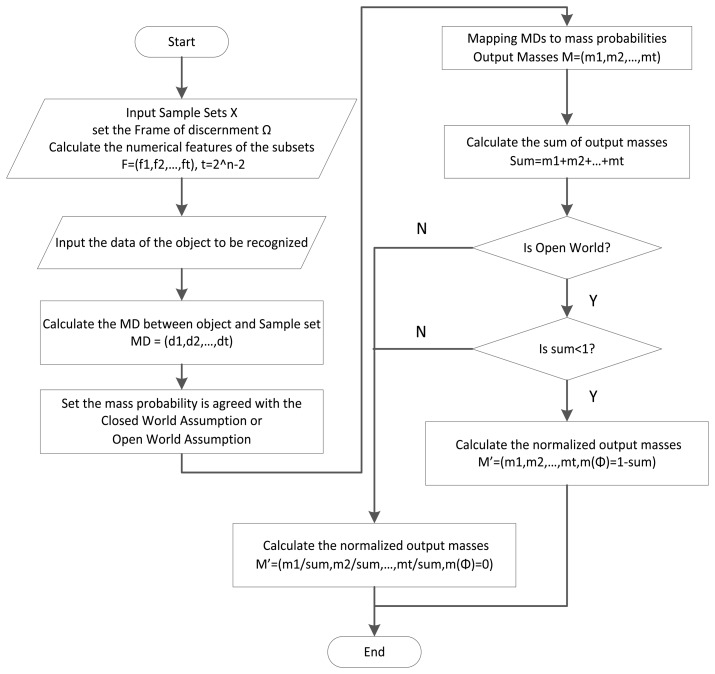
Process of proposed mass constructing method.

Figure 6 illustrates the assignment process for the masses in DSET. The detailed description of the process is as follows:

(1) This first step is calculating the statistical features of each subset in 2^Ω^, especially the compound class of the power set. In order to guarantee the accuracy, it requires us to input adequate sample data to get correct statistical features.
(2) Subsequently is the calculation of the sample set's numerical features. After the beginning of the algorithm, the frame of the discernment should be set up according to the specific situation. All the possible subsets (proposition sets) are constructed, and the mean value and standard deviation of every subset are computed.
(3) Then Compute the MDs between the object and the classes of2^Ω^. For all classes where the mean and standard deviation values exist, calculate the MDs with the data collected from the observed object.
(4) Transfer MDs to the masses. With the use of the transfer function, the obtained MDs will be converted into masses.
(5) After the mapping step, the output is not the final answer we want. It should be normalized under the Closed World Assumption (CWA) or Open World Assumption (OWA). In CWA, the object to be recognized must belong to one of the subsets in Ω, which means the mass of the null set is 0, that is *m*(Ø) = 0. In OWA, unknown classes are allowed to exist, and the mass of the null set may be larger than 0. In this situation, if the sum of all the masses is larger than 1, which means the mass of the known classes is large enough, the mass of the null set should be set to 0, otherwise, *m*(Ø) = 0 1 − *sum*.

## 6. Experimental Results

### 6.1. Setup of Transformer Fault Diagnosis

A transformer is an important distribution component in a power system. The security and reliability of the power system is heavily influenced by the transformer. In order to accurately and effectively detect the type of fault in a transformer, different sensors are applied in the inner space of the transformer [23,24], like gas sensor, voltage sensor, temperature sensor and humidity sensor. Now, we use DSET to solve the problem because DSET's advantage is fusing multisource data into one unified result. Here we use the method to construct masses form data collected by the gas sensors as a validation of the proposed method.

There are various kinds of gases in the transformer's internal space. In this paper, we use H_2_, CH_4_, C_2_H_6_, C_2_H_4_ and C_2_H_2_ as the basis of the diagnosis. When different faults occur, the percentage of each gas will change. To simplify the experiment, we consider three types of states: the normal state (No), temperature fault (Te), and discharge fault (Di). The collected data are the percentages of each gas for a total number of 600 pieces of data (120 samples). In this case, the frame of discernment is Ω = {No, Di, Te}, and the power set is 2^Ω^ = {No,Di,Te,No∩Di,No∩Te, Di∩Te,No∩Di∩Te}, except the null set.

### 6.2. Experiment Results

**Figure 7.**
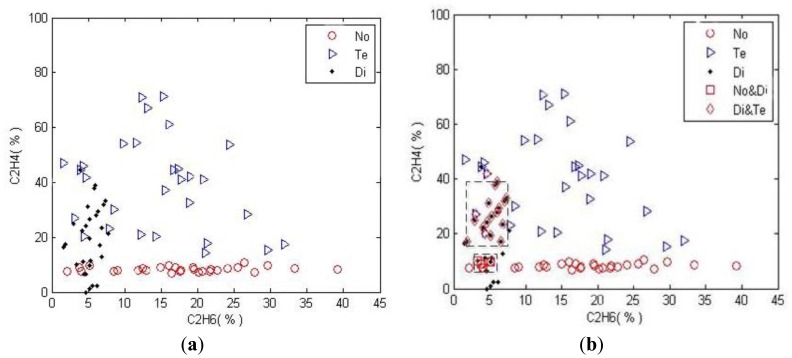
The distribution of the sample data, which includes (**a**): the original samples collected in the 3 conditions; (**b**): the all calculated sample sets of the power set.

To illustrate the proposed algorithm, we take the combination of two gases as a sample; they are (C_2_H_6_, C_2_H_4_). There are 40 samples for each state. To examine the proposed algorithm, 30 samples for each state are used as the basis of the classification, and the remaining 10 samples are used as the validation data. Before the process of finding out the scope of the compound class, some outlier data should be eliminated, because they will decrease the accuracy of the algorithm. Here, we delete the three samples with the largest MDs to form a crisp sample set. Figure 7 shows the distribution map of (C_2_H_6_, C_2_H_4_) in different fault conditions.

**Figure 8.**
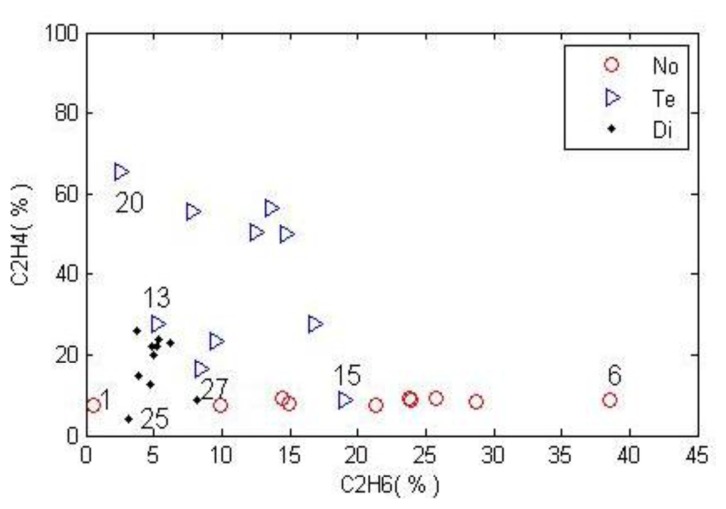
The distribution map of the validate data.

In Figure 7, the circle, triangle, and hexagon represent the normal state (No), temperature fault (Te), and discharge fault (Di), respectively. The first step is to determine the sample sets for all subsets in 2^Ω^ In order to simplify the computation complexity, we calculate the intersection scope by rectangular area. After the process of calculating the intersection scopes, the intervals of the compound subsets are No∩Di = ([2.15,7.70][6.93,10.63]), Di∩Te=([1.63,7.70][14.19,44.37]) , No∩Te = *ϕ*, No∩Di∩Te = *ϕ* and the samples of each subset in 2^Ω^ are depicted in Figure 7b, No&Te and No&Te&Di are not shown in the map as they equal to the null set. The validate data and the corresponding results are described in Figure 8. Here *λ*_min_, *λ*_max_ are set as 0.001,0.999, respectively.

**Figure 9.**
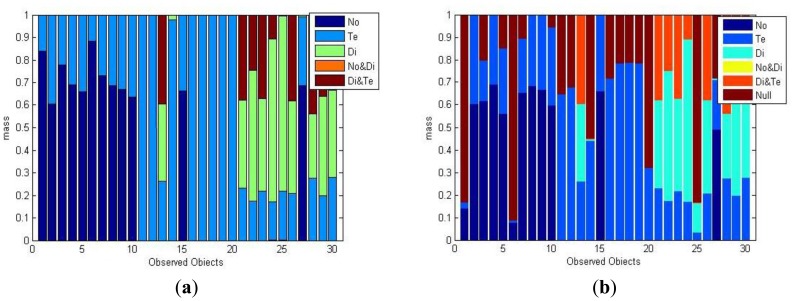
The obtained masses from the validate data. (**a**) masses obtained under CWA; (**b**) masses obtained under OWA.

As shown in Figure 8, new observed fault data is collected to validate the correctness of the proposed algorithm. The distribution map shows the 30 validate samples collected in the three conditions. The corresponding masses of the validate samples are shown in Figure 9. The horizontal axis denotes the number of validate sample, vertical axis is the mass assignments of each sample and the sum of each mass equals to 1. The first 10 samples were collected when the transformer was normal, and 10–20 were collected when the transformer was in a temperature fault state, the last 20–30 samples correspond to the state of a discharge fault. After the process of the proposed method, masses under CWA and OWA are obtained, as shown in Figure 9a,b, respectively.

Apparently, most of the masses under CWA are correct, except Nos.13, 15, 27. These are incorrect because boundaries between samples are not clear and the three objects' positions are too far from the sets they should belong to, which causes them to lie in the scope of other samples. A good way to get an optimized mass result is to calculate the MD by higher dimensional data pattern, such as (C_2_H_6_, C_2_H_4,_ H_2_). In OWA, unknown states of the transformer are allowed. Thus, the null set's mass may be larger than 0. The belief assignment of the null set in Nos. 1, 6, 14, 20, and 25 are obviously larger than the other masses. These results predict that there are maybe unknown fault types unknown in the frame of discernment. However, considering the researchers have had a comprehensive understanding of the transformer's fault conditions, hence the world is better to be set as “closed” in this situation to get a more accurate classify result.

**Figure 10.**
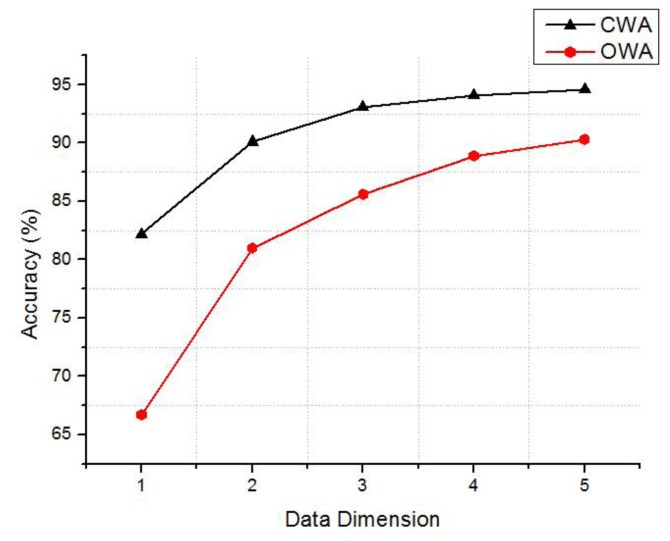
Average accuracies of the masses calculated with different dimensional data

To verify the accuracy, more tests are conducted with different multi-dimensional gas data patterns from 1 to 5. In 1-dimension, only one gas data is used to construct masses, such as C2H4, in 2-dimensions, two gas data are combined to construct masses, like (C2H6, C2H4). It is the same with 3- to 5- dimensional data patterns. We define the accuracy as:

(17) $$r=\frac{\sum_{i}^{c}N_{i}}{N}$$

where *r* is the accuracy rate, *N* is the total number of objects, *N_i_* is the number of objects which have been correctly classified from its mass. If an object belongs to class *ω_i_*, it is correctly classified by satisfying *m*(*ω_i_*) = max{*m*(*ω*_1_),…, *m*(*ω_c_*)}. The tests results are mean value of all possible data patterns in different dimensions. For example, the test accuracy of one-dimensional data is the average of the accuracies of the five patterns: (H_2_), (CH_4_), (C_2_H_6_), (C_2_H_4_) and (C_2_H_2_). The accuracies in CWA and OWA are illustrated in the Figure 10. The accuracies of the constructed masses increase with the increasing of the test data's dimension, especially from 1-dimension to 3-dimensions. Apparently, a higher dimension of the data will be beneficial to get more accurate masses. In practice, it is better not obtain mass from 1-dimensional data. In conclusion, the results illustrates that calculating belief assignment in DSET by MD is completely feasible and the method is easy to implement in sensor nodes.

## 7. Discussions and Conclusions

In the proposed algorithm, there are a couple of caveats that should be observed. First, it should be emphasized that the sample data used in the proposed algorithm should be adequate enough to get the correct statistical features of the sample data. This is a disadvantage compared to other method like ANN, which require low amount data to train the network. In Section 4, the calculation process of the intersection classes' scope was developed. The boundaries of intersection scopes calculated by the proposed method are straight lines, in reality they maybe irregular curves, which means the calculated intersections are approximations and may not that accurate. However, in practice, finding out the exact intersection space is a tough problem and there is no significance in sacrificing large amount computations in calculating the exact intersection scopes. Hence we choose the proposed method to calculate intersections, it is fast and efficiency and its experimental accuracy results are acceptable, too.

The algorithm presented in this paper is helpful in dealing with the multisource data of a WSN. In a WSN, the sensor nodes do not have an enormous amount of computing ability and their energy is limited. It is very meaningful to fuse the multisource data before uploading to the servers, which releases the transmission pressure of the sink node. The proposed paradigm has a high calculation speed, and the output masses are reasonable and stable, which lays a good foundation for the subsequent fusion calculation steps. We believe the paradigm proposed in this paper has a promising future in application. The future work may include the following: (1) applying the proposed algorithm in fuzzy set theory as the method to calculate the membership; (2) finding another way to compute the intersection between crisp focal elements in DSET and verifying its reasonableness; (3) developing a flexible and effective neural network by DSET and MD and examining its performance; (4) finding a reasonable way to calculate the MDs from multimedia data, rather than just scalar data.
